# Does interdisciplinary breadth shape research data sharing and citation impact? Evidence from six research fields

**DOI:** 10.3389/frma.2026.1952277

**Published:** 2026-09-24

**Authors:** Liu Xu, Huan Chen

**Affiliations:** School of Information Management, Central China Normal University, Wuhan, China

**Keywords:** data sharing, interdisciplinary breadth, open data citation advantage, open science, scientometrics

## Abstract

**Introduction:**

Research data sharing has become an important component of open science, yet sharing benefits vary across research contexts. Existing studies have examined factors influencing data sharing and the citation advantage associated with open data, but less is known about how interdisciplinary breadth relates to observable sharing practices and whether the citation advantage of sharing differs across disciplines. This study investigates the relationships among interdisciplinary breadth, observable sharing practices, and citation impact.

**Methods:**

The study uses 566,188 journal articles indexed in OpenAlex from 2020 to 2025. Interdisciplinary breadth was operationalized as the number of Level-0 disciplinary concepts associated with each article. Observable sharing practices were identified through repository links, supplementary-material links, and textual sharing statements. Regression models were applied to examine associations among interdisciplinary breadth, data sharing, and citation impact across six research fields, and BERTopic-based topic modeling was also applied in history and digital humanities to explore topic-level variations.

**Results:**

The results show that interdisciplinary breadth had a predominantly negative but non-linear association with observable sharing, with sharing probability generally declining as breadth increased, although the decline weakened at higher levels. Articles with observable sharing signals were associated with higher citation counts overall, but the magnitude of this association varied substantially across fields. History exhibited the largest positive citation advantage, whereas digital humanities showed a negative but statistically uncertain association. Topic-level analysis further revealed that observable sharing was concentrated in specific research topics with distinct methodological and infrastructural characteristics.

**Discussion:**

These findings highlight that openness practices and their scholarly returns are influenced by disciplinary contexts. Understanding research data sharing therefore requires consideration of both researchers' willingness to share and the infrastructures and evaluation systems embedded in different research disciplines.

## 1 Introduction

Open science has increasingly reshaped the production, communication, and evaluation of scholarly knowledge. Among its core practices, research data sharing has attracted growing attention because research data serve as evidence supporting published findings and as valuable scholarly outputs that facilitate verification, replication, and reuse (Walters, 2020). The FAIR (Findable, Accessible, Interoperable, and Reusable) guiding principles for scientific data management and stewardship emphasize that research data should be findable, accessible, interoperable, and reusable to maximize their scientific value and promote responsible data stewardship (Wilkinson et al., 2016). Prior research has suggested that making research data available can increase scholarly visibility (Reales and Wallace, 2023). Accordingly, previous studies have identified various factors influencing researchers' willingness to share data, such as perceived benefits, concerns about misuse, data management costs, institutional pressures, and repository infrastructures (Lortie, 2021; Jeng and He, 2022). However, these studies have mainly focused on individual-level motivations and organizational conditions, while paying less attention to how characteristics of the research itself may shape the feasibility and visibility of data-sharing practices. This leaves open the question of how characteristics of research content, such as interdisciplinary breadth, may shape sharing practices and whether the scholarly returns associated with sharing vary across research contexts.

We conceptualize disciplinary characteristics in terms of interdisciplinary breadth, defined as the extent to which an article draws on concepts from multiple disciplines (Ponzi, 2002). Here, interdisciplinary breadth refers to the range of disciplinary concepts represented in an article and reflects the disciplinary diversity of its research content. It captures the knowledge characteristics embedded in a publication rather than collaboration among researchers or organizations. Research articles vary in the range of disciplinary knowledge they incorporate, and this variation may affect both the value and the complexity of sharing their underlying data. Existing studies have reported substantial differences in data availability and sharing behaviors across research fields (Tenopir et al., 2020; Tedersoo et al., 2021; Khan et al., 2023). For instance, in fields characterized by standardized data structures and well-established repositories, such as areas of the natural sciences, data sharing has become increasingly embedded in routine research workflows. By contrast, research in the humanities and social sciences often involves contextualized, qualitative, interpretive, or ethically sensitive materials, which may require more complex approaches to documentation, preservation, and access (Zuiderwijk et al., 2020; Strange et al., 2023). Articles with greater interdisciplinary breadth may face additional challenges because they can involve heterogeneous data types, methodological conventions, and documentation standards, which may influence the development of common sharing practices across disciplinary boundaries. Nevertheless, whether interdisciplinary breadth is associated with observable sharing practices remains poorly understood.

The relationship between interdisciplinary breadth and data sharing represents only one part of this issue. Beyond understanding whether research characteristics influence sharing practices, it is also important to examine whether the scholarly returns associated with sharing differ across disciplinary contexts. The open data citation advantage (ODCA) refers to the observation that articles linked to accessible research data tend to receive more citations than comparable articles without available data (Piwowar and Vision, 2013). Previous studies have provided evidence for positive associations between research data sharing and citation impact (Colavizza et al., 2020, 2024), suggesting that shared data may enhance research visibility by improving transparency, enabling reuse, and expanding scholarly connections. However, existing evidence has mainly focused on fields with relatively mature data infrastructures, and less is known about whether the magnitude of ODCA varies across research fields with different publication cultures, research practices, and data characteristics. Examining interdisciplinary breadth, sharing practices, and citation impact together can therefore provide a broader view of how research characteristics and disciplinary contexts relate to both sharing and scholarly visibility.

Moreover, field-level comparisons may not fully explain such variation. Research topics within the same field can differ substantially in their methodological orientations, data requirements, ethical constraints, and potential for reuse. For example, digital humanities research may involve diverse forms of digital collections, cultural heritage data, and computational methods, creating different opportunities and challenges for data sharing (Higgins et al., 2024; Belteki et al., 2025). Aggregate disciplinary patterns may therefore conceal considerable within-field heterogeneity. Understanding variation in data sharing and its citation advantage consequently requires field-level comparison together with closer examination of topic-level patterns within fields. Topic-level analysis can further examine such disciplinary heterogeneity by revealing how sharing and citation patterns vary across research topics within the same field.

Against this background, this study investigates the relationships among interdisciplinary breadth, observable sharing practices, and citation impact across six research fields: biology, physics, history, sociology, bioinformatics, and digital humanities. In the empirical analysis, observable sharing is identified from publicly available evidence of research data or code availability. Because data-sharing records constitute the large majority of identified sharing cases, research data sharing remains the primary focus of the study, while the composition of the sharing indicator is reported explicitly in the methods. Specifically, this study addresses the following three research questions:

RQ1: How is interdisciplinary breadth associated with observable sharing practices?

RQ2: Is observable sharing associated with citation advantages, and how does this association vary across research fields?

RQ3: How do topic-level observable sharing patterns differ within research fields exhibiting contrasting citation advantages?

Using these research questions as a framework, this study analyzes 566,188 journal articles indexed in OpenAlex between 2020 and 2025. Regression analyses examine the associations between interdisciplinary breadth, observable sharing, and citation impact across six research fields, while BERTopic is used as an exploratory topic-level analysis in history and digital humanities to examine within-field topic heterogeneity.

## 2 Background and related research

### 2.1 Interdisciplinarity and interdisciplinary breadth of research

Research on interdisciplinarity has primarily examined its conceptualization, measurement, and implications for research processes and outcomes. Porter et al. (2007) proposed that the interdisciplinarity of research activities could be measured using the disciplinary distribution and integration of cited references. Drawing on diversity theory, Stirling proposed three dimensions of diversity—variety, balance, and disparity—which provided the theoretical foundation for the subsequent application of indicators such as Rao-Stirling diversity (Stirling, 2007). Through long-term comparisons across multiple research fields, Porter and Rafols (2009) found that science as a whole has shown an increasing trend toward interdisciplinarity, although pathways of knowledge integration vary across fields.

Wagner et al. (2011) argued that interdisciplinarity involves both the coexistence of different disciplinary elements and the depth of knowledge integration. Therefore, it should be assessed through a combination of quantitative indicators and process-oriented understanding. These studies also indicate that interdisciplinarity is multidimensional (Stirling, 2007). In the present study, interdisciplinary breadth refers specifically to the range of disciplinary concepts represented in an article and should not be interpreted as direct evidence of collaboration among authors, institutions, or disciplinary teams. In contrast to studies focusing on collaborative structures among researchers or organizations, this study focuses on article-level disciplinary breadth as a characteristic of research content.

Prior research has also highlighted both the potential benefits and costs of crossing disciplinary boundaries. Interdisciplinary research may generate greater visibility and broader impact, but cognitive, coordination, and dissemination costs can increase as disciplinary distance grows (Yegros-Yegros et al., 2015; Leahey et al., 2017; Hall et al., 2018). These findings suggest that disciplinary heterogeneity can create both opportunities and additional coordination demands. Although previous studies have mainly examined the relationship between interdisciplinarity and research outcomes, they provide a conceptual basis for examining whether article-level disciplinary breadth may also be associated with research practices such as data sharing.

### 2.2 Research on data sharing and citation advantage

Research data sharing is an important practice within open science, and its potential relationship with academic impact has frequently been examined in studies of the open data citation advantage (ODCA). ODCA refers to the observed tendency for articles associated with accessible research data to receive more citations than comparable articles without accessible data. A study in the life sciences found that articles associated with publicly available research data generally received more citations (Piwowar and Vision, 2013). Based on a larger-scale dataset, Colavizza et al. (2020) further demonstrated that explicit links between publications and research datasets were significantly associated with higher citation performance. Subsequently, research attention expanded from data sharing to various open practices, including data, code, and preprints. Existing studies have shown that depositing data in online repositories is generally associated with higher citation counts, although the magnitude of this association is weaker than that observed for preprints, and substantial disciplinary differences exist (Colavizza et al., 2024).

However, existing research on citation advantages still has several limitations. First, much of the available evidence comes from the life sciences, highly influential multidisciplinary journals, or research environments with relatively mature open policies. Therefore, the applicability of these findings to the social sciences, humanities, and emerging interdisciplinary fields requires further investigation. Second, in fields such as digital humanities, although datasets, databases, code, and visualization outputs have become increasingly important, the evaluation of these research outputs remains uneven, which may weaken the translation of open practices into gains in academic reputation (All European Academies, 2023). Therefore, the open data citation advantage is likely shaped jointly by disciplinary norms, infrastructure development, and the maturity of evaluation systems.

## 3 Materials and methods

### 3.1 Study design and data source

This study adopted a data-driven research design that combined article-level bibliometric analysis with topic-level semantic analysis. At the article level, bibliographic metadata and citation information were used to examine the relationship between interdisciplinary breadth and observable sharing practices and to estimate the citation advantage associated with data sharing. At the topic level, a deep-learning-based topic modeling approach was applied to explore variations in data-sharing practices across research topics within selected disciplines. Together, these analyses provided both disciplinary-level and topic-level perspectives on data-sharing patterns.

The bibliographic data were obtained from OpenAlex, an open scholarly knowledge graph that indexes more than 250 million scholarly works. OpenAlex provides a hierarchical concept classification system ranging from Level 0 to Level 5, which supports the measurement of interdisciplinary breadth at the article level. Its metadata also include abstract inverted indexes and links to external or full-text resources, enabling the joint analysis of structured metadata and unstructured textual information. Bibliographic metadata and citation counts used in this study were retrieved on 16 March 2026. Because an article could be retrieved under more than one target concept, duplicate OpenAlex IDs across field-specific retrievals were collapsed so that each article appeared only once in the analytical sample. When duplicate records occurred, the first record in the fixed data-collection order, including its discipline label, was retained.

### 3.2 Sample construction and disciplinary classification

To compare data-sharing practices across different disciplinary environments, six research fields were selected and categorized into three groups. The selection was guided by the observation that research fields differ substantially in data characteristics, sharing practices, and disciplinary traditions. Therefore, the selected fields were intended to provide contrasting research contexts rather than represent all disciplinary domains. Previous studies have shown substantial disciplinary differences in data production, sharing, and reuse, including differences between fields within the same broad disciplinary domain (Pujol Priego et al., 2022; Khan et al., 2023). Physics and biology were therefore selected as two established natural science fields with different data practices, while history and sociology represented different research traditions within the humanities and social sciences. Bioinformatics and digital humanities were selected as fields in which computational approaches are closely integrated with established disciplinary traditions; the growing use of large-scale digital resources and quantitative methods in humanities research further supports the inclusion of digital humanities (Michel et al., 2011). Two fields were included in each group to permit both within-group and across-group comparisons.

Data were collected through the OpenAlex application programming interface using Python scripts. The observation period covered the period from 1 January 2020 to 31 December 2025. For each target field, the corresponding OpenAlex Concept ID was identified, and articles containing that concept in their OpenAlex concept metadata were retrieved. Field assignment was based on OpenAlex concept metadata rather than author affiliations or journal categories. Because OpenAlex can assign multiple concepts to the same article, duplicate records were removed at the article-ID level before statistical analysis. For each discipline and publication year, journal articles were ranked in descending order by OpenAlex citation counts, and up to 20,000 articles were retrieved. The same upper limit was applied to each discipline-year stratum to reduce large differences in sample size across fields and years and to maintain a manageable dataset for article-level and text-based analyses. Records without a DOI, retracted publications, and records without abstracts were excluded. After data collection, screening, and cleaning, the final dataset contained 566,188 journal articles. The final sample comprised 106,199 articles in biology, 120,000 in physics, 115,511 in history, 109,254 in sociology, 113,301 in bioinformatics, and 1,923 in digital humanities.

### 3.3 Variable measurement

#### 3.3.1 Identification of data-sharing practices

Data-sharing practices were identified using a three-level procedure that combined structured metadata links with unstructured text mining. At the first level, repository-based sharing was identified by matching URLs in the publication metadata against a predefined controlled list of major third-party repositories, comprising Zenodo, Figshare, Dryad, OSF, Dataverse, Mendeley Data, Kaggle, PANGAEA, GitHub, GitLab, Bitbucket, Hugging Face, and SourceForge. Repositories primarily associated with data and those primarily associated with code were recorded separately during identification. A publication was classified as containing first-level sharing evidence when at least one of its metadata links pointed to a repository included in the controlled list.

At the second level, supplementary-material sharing was identified through standardized URL patterns indicating that research materials had been deposited on a journal server or provided as supplementary files associated with the article. The main URL patterns contained terms such as “supplementary,” “supporting-information,” and “suppl_data.”

At the third level, semantic sharing statements were identified from unstructured text. Regular expressions and semantic rules were applied to article abstracts and available data availability statements to detect phrases indicating that data or code had been made available, such as “Data available at” and “Code provided in.” The rules also covered expressions containing terms such as “data,” “dataset,” “code,” or “script” combined with availability, provision, or deposition statements and repository URLs.

A binary variable, *Share*_*i*_, was constructed to represent observable sharing practices. It was coded as 1 when article i satisfied at least one of the three identification criteria and 0 otherwise. The identification procedure captured publicly observable evidence of both data and code availability. Data-sharing and code-sharing records were distinguished during classification, while the binary indicator *Share*_*i*_ was used to represent observable sharing in the statistical analysis. The observable sharing rate was calculated as the number of articles with observable sharing evidence divided by the total number of articles assigned to that topic.

#### 3.3.2 Measurement of interdisciplinary breadth

To quantify interdisciplinary breadth, this study used the number of Level-0 concepts associated with each article in OpenAlex. Following the variety dimension of diversity (Stirling, 2007) and prior bibliometric approaches to interdisciplinarity (Porter et al., 2007; Wagner et al., 2011), the number of distinct Level-0 concepts was used as an article-level measure of disciplinary breadth. The variable *Diversity*_*i*_ was defined as the count of unique Level-0 concepts associated with article i. A higher *Diversity*_*i*_ indicates that an article integrates knowledge from a broader range of disciplinary domains. This measure captures the disciplinary breadth of article content; collaboration among authors, institutions, or disciplinary teams falls outside its scope.

#### 3.3.3 Measurement of citation impact

Citation counts were used as a proxy for academic impact (Bornmann and Daniel, 2008). Because citation distributions are typically highly skewed and include zero-citation observations, citation counts were log-transformed as follows Thelwall (2016):


$$\begin{array}{l} Y_{i}={\text{ln}(\text{Citation}\text{s}}_{i+1}) \end{array}$$ (1)

### 3.4 Statistical analysis

Descriptive analyses were conducted to examine disciplinary and temporal differences in data-sharing rates. The subsequent statistical analysis consisted of two components. First, non-linear regression analysis was used to examine the relationship between interdisciplinary breadth and data-sharing practices. Second, ordinary least squares (OLS) regression models were used to estimate the citation advantage associated with data sharing and to examine disciplinary heterogeneity in this advantage. For the regression analyses, *Year*_*centered*_*i*_ was calculated as publication year minus 2020, so that values ranged from 0 for articles published in 2020 to 5 for those published in 2025.

#### 3.4.1 Non-linear relationship between interdisciplinary breadth and data sharing

As a preliminarily examination of the relationship between interdisciplinary breadth and data-sharing practices, articles were grouped according to their *Diversity*_*i*_. For each diversity-score group, the mean data-sharing rate and its 95% confidence interval were calculated.

A quadratic polynomial curve was fitted to the grouped means to visualize the overall relationship and identify potential non-linear patterns between interdisciplinary breadth and data-sharing practices. A quadratic specification was adopted because the relationship between interdisciplinarity and research outcomes may not follow a simple linear pattern. Previous studies have shown that different dimensions of interdisciplinarity can exhibit curvilinear relationships with research impact, suggesting that the effects of increasing disciplinary breadth may vary across different levels of integration (Yegros-Yegros et al., 2015). This specification quadratic specification was then used as a parsimonious approach to examine whether the association between interdisciplinary breadth and observable sharing changed systematically across levels of breadth (Cudeck and du Toit, 2002). Similar non-linear specifications have been used in previous interdisciplinarity research to examine whether the effects of disciplinary diversity vary across different levels of integration (Yegros-Yegros et al., 2015; Wang et al., 2022). The relationship was estimated using the following linear probability model (LPM):


$$\begin{array}{l} \text{Shar}{\text{e}}_{\text{i}}=\alpha +\beta_{1}\text{Diversit}{\text{y}}_{\text{i}}+\beta_{2}{\text{Diversit}{\text{y}}_{\text{i}}}^{2}\\ \;+\gamma {Year\text{\_}centered}_{i}+\mu_{d}+\varepsilon_{i} \end{array}$$ (2)

where *Share*_*i*_ represents the observable sharing status of article *i*; *Diversity*_*i*_ represents interdisciplinary breadth; ${Diversity_{i}}^{2}$ captures potential non-linear effects; *Year*_*centered*_*i*_ controls for publication-year differences; and μ_*d*_ represents discipline fixed effects.

A statistically significant coefficient of the quadratic term indicates the presence of a non-linear relationship between interdisciplinary breadth and data-sharing practices. The combination of the linear and quadratic coefficients was used to determine the direction of the relationship and the location of the estimated turning point. To ensure the robustness of statistical inference, heteroskedasticity-consistent standard errors (HC3) were used (MacKinnon and White, 1985). The LPM was retained as the main specification because its coefficients can be interpreted directly on the probability scale (Hellevik, 2009). A logistic regression using the same explanatory variables was also estimated as a robustness check, and average marginal effects (AMEs) were calculated to facilitate comparison with the LPM estimates.

#### 3.4.2 Citation advantage models

To examine whether observable sharing practices were associated with citation impact, ordinary least squares (OLS) regression models were estimated (Thelwall, 2016). The baseline model was specified as follows:


$$\begin{array}{l} {\text{Y}}_{\text{i}}=\alpha +\beta_{1}\text{Shar}{\text{e}}_{\text{i}}+\beta_{2}\text{Diversit}{\text{y}}_{\text{i}}\\ \;+\gamma Year\text{\_}centered_{i}+\mu_{d}+\varepsilon_{i} \end{array}$$ (3)

where *Y*_*i*_ represents the log-transformed citation count of article *i*. The coefficient β_1_ captures the average citation difference associated with data-sharing practices after controlling for interdisciplinary breadth, publication year, and discipline, while β_2_ represents the association between interdisciplinary breadth and citation impact. The percentage citation difference associated with sharing was calculated as$\left(e^{\beta_{1}}-1\right)\times 100$ (Halvorsen and Palmquist, 1980).

To examine whether the citation advantage of data sharing varies across disciplines, discipline-specific analyses were further conducted. All regression models used heteroskedasticity-robust standard errors (HC3) to ensure reliable statistical inference. The following model was estimated separately for each discipline:


$$\begin{array}{l} {\text{Y}}_{\text{i}}=\alpha_{d}+\beta_{d}\text{Shar}{\text{e}}_{\text{i}}+\theta_{\text{d}}\text{Diversit}{\text{y}}_{\text{i}}\\ \;+\gamma_{\text{d}}{Year\text{\_}centered}_{i}+\varepsilon_{i},i\in d \end{array}$$ (4)

where *d* denotes each of the six disciplines and β_*d*_ represents the discipline-specific association between observable sharing and citation impact. Two-sided *p*-values and 95% confidence intervals were reported for the regression estimates.

Because articles published in 2025 had a shorter period in which to accumulate citations, the overall citation model and the six discipline-specific models were additionally re-estimated after excluding articles published in 2025. This sensitivity analysis assessed the robustness of the estimates to the shorter citation window of the most recent publication cohort.

### 3.5 Topic modeling with BERTopic

To further investigate variations in data-sharing practices across research topics within a field, this study employed BERTopic for topic modeling. Compared with traditional probabilistic topic models such as Latent Dirichlet Allocation (LDA), BERTopic incorporates contextualized language representations and is capable of generating semantically coherent and fine-grained topic representations (Grootendorst, 2022). The topic modeling analysis was applied to all articles in the history and digital humanities samples. These two fields were selected because they exhibited contrasting associations between data sharing and citation impact in the discipline-specific regression analyses. The topic-level analysis was designed as an exploratory follow-up to the discipline-specific analyses, examining how sharing and citation patterns varied across topics within the two fields.

The BERTopic procedure consisted of three sequential stages. First, a pretrained Transformer model was applied to convert article abstracts into high-dimensional embeddings that captured contextual semantic information. Second, Uniform Manifold Approximation and Projection (UMAP) was used to reduce the dimensionality of the embeddings, and Hierarchical Density-Based Spatial Clustering of Applications with Noise (HDBSCAN) was subsequently applied to identify clusters of semantically similar documents based on density patterns. Third, class-based term frequency-inverse document frequency (c-TF-IDF) was used to extract representative terms from each cluster, which were then used to generate semantic labels for the identified topics (Grootendorst, 2022).

The resulting topic distributions, together with topic-level observable sharing rates and citation characteristics, were used to construct topic-level scatterplots illustrating how data-sharing practices varied across research topics. These topic-level patterns were used to characterize within-field heterogeneity in history and digital humanities.

## 4 Results

### 4.1 Sample characteristics

The final dataset comprised 566,188 journal articles published between 2020 and 2025 across six research fields. Table 1 presents the distribution of the sample, the mean citation count, and the mean interdisciplinary breadth for each field. Physics had the largest sample, with 120,000 articles, followed by history with 115,511 articles, bioinformatics with 113,301 articles, sociology with 109,254 articles, and biology with 106,199 articles. Digital humanities had a substantially smaller sample of 1,923 articles. Marked differences were observed in citation counts across fields. Biology had the highest mean citation count at 123.58, followed by physics at 103.57 and sociology at 49.99. History and bioinformatics had mean citation counts of 12.68 and 13.90, respectively, whereas digital humanities had the lowest mean citation count at 1.42.

**Table 1:** Descriptive statistics by discipline.

Group	Discipline	*N*	Mean of citations	Mean of diversity
Natural sciences	Biology	106,199	123.58	3.06
Natural sciences	Physics	120,000	103.57	3.63
Humanities social	History	115,511	12.68	4.11
Humanities social	Sociology	109,254	49.99	4.17
Interdisciplinary	Bioinformatics	113,301	13.9	2.34
Interdisciplinary	Digital humanities	1,923	1.42	3.69
Total	All	566,188	60.15	3.47

The mean interdisciplinary breath varied across fields. Sociology had the highest mean interdisciplinary breadth at 4.17, followed by history at 4.11, digital humanities at 3.69, physics at 3.63, and biology at 3.06. Bioinformatics had the lowest mean score at 2.34. These descriptive results indicate that fields commonly described as interdisciplinary did not necessarily have the highest number of Level-0 concepts assigned to their publications.

### 4.2 Disciplinary and temporal patterns of data sharing

A total of 16,811 articles were identified as having observable sharing evidence, including 15,960 data-sharing records and 851 code-sharing records. Data-sharing records accounted for the large majority of identified cases. The observed field-level pattern therefore showed a clear concentration of data sharing in physics, whereas the other five fields all had sharing rates below 2.5%. Within the humanities and social sciences, digital humanities had a slightly higher sharing rate than history and sociology.

Figure 1 shows the mean data-sharing rate for each of the six fields during the observation period. Physics had the highest sharing rate, at 6.17%, substantially exceeding the rates observed in the other fields. Digital humanities and bioinformatics had sharing rates of 2.32% and 2.01%. History and sociology both had rates of approximately 1.94%, while biology had a sharing rate of 1.90%. Figure 2 presents the temporal evolution of data-sharing rates from 2020 to 2025. Data-sharing rates showed different temporal trajectories across disciplines, with most fields exhibiting a downward pattern during the observation period. However, the magnitude and timing of these changes varied substantially across disciplines. Physics maintained the highest sharing rate but experienced the most pronounced decline, whereas biology and bioinformatics showed relatively gradual reductions. In contrast, history, sociology, and digital humanities exhibited consistently lower sharing rates with noticeable annual fluctuations. These patterns represent temporal variations in the proportion of articles reporting accessible data. They reflect changes in reporting practices, while the absolute volume of shared research materials cannot be directly inferred from these results.

**Figure 1:**
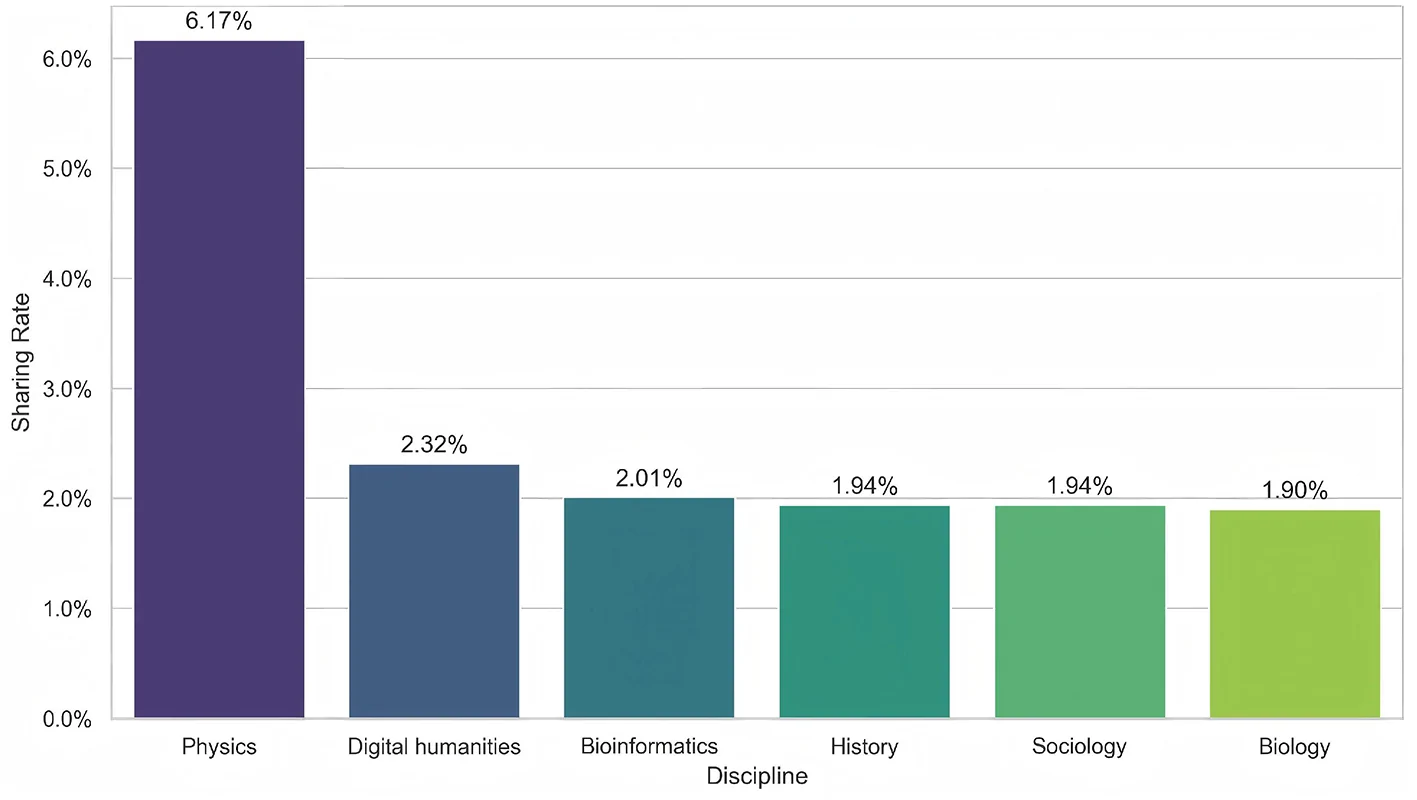
Observable sharing rates across disciplines.

**Figure 2:**
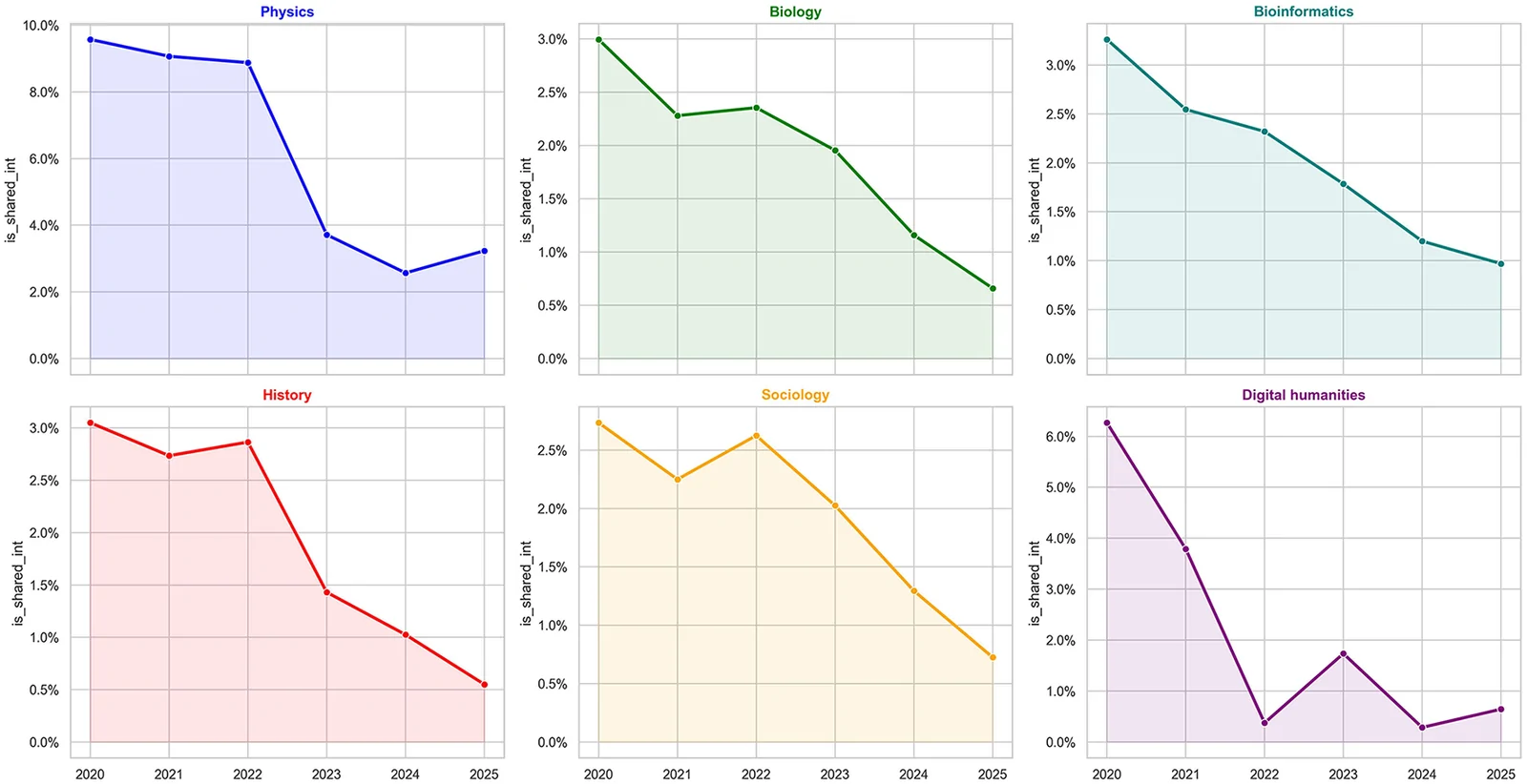
Temporal trends in data-sharing rates by discipline, 2020–2025.

### 4.3 Interdisciplinary breadth and observable sharing

Figure 3 shows the relationship between *Diversity* and the mean data-sharing rate. The descriptive pattern indicates that data-sharing rates generally declined as the number of Level-0 concepts associated with an article increased. Articles associated with one to three Level-0 concepts had a mean sharing rate of approximately 3.43%. In this range, the fitted curve remained comparatively high, and the confidence intervals were relatively narrow. By contrast, articles associated with five to eight or more Level-0 concepts had a mean sharing rate of ~1.74%. The sharing rate in the high-breadth group was therefore approximately half that observed among articles with lower interdisciplinary breadth. The fitted quadratic curve showed a predominantly downward pattern, indicating that broader disciplinary coverage was associated with a lower probability of observable data sharing. At the upper end of the *Diversity* distribution, particularly when the score approached 10, the confidence intervals became wider. The number of observations also declined substantially at these higher scores, increasing the uncertainty of the corresponding sharing-rate estimates.

**Figure 3:**
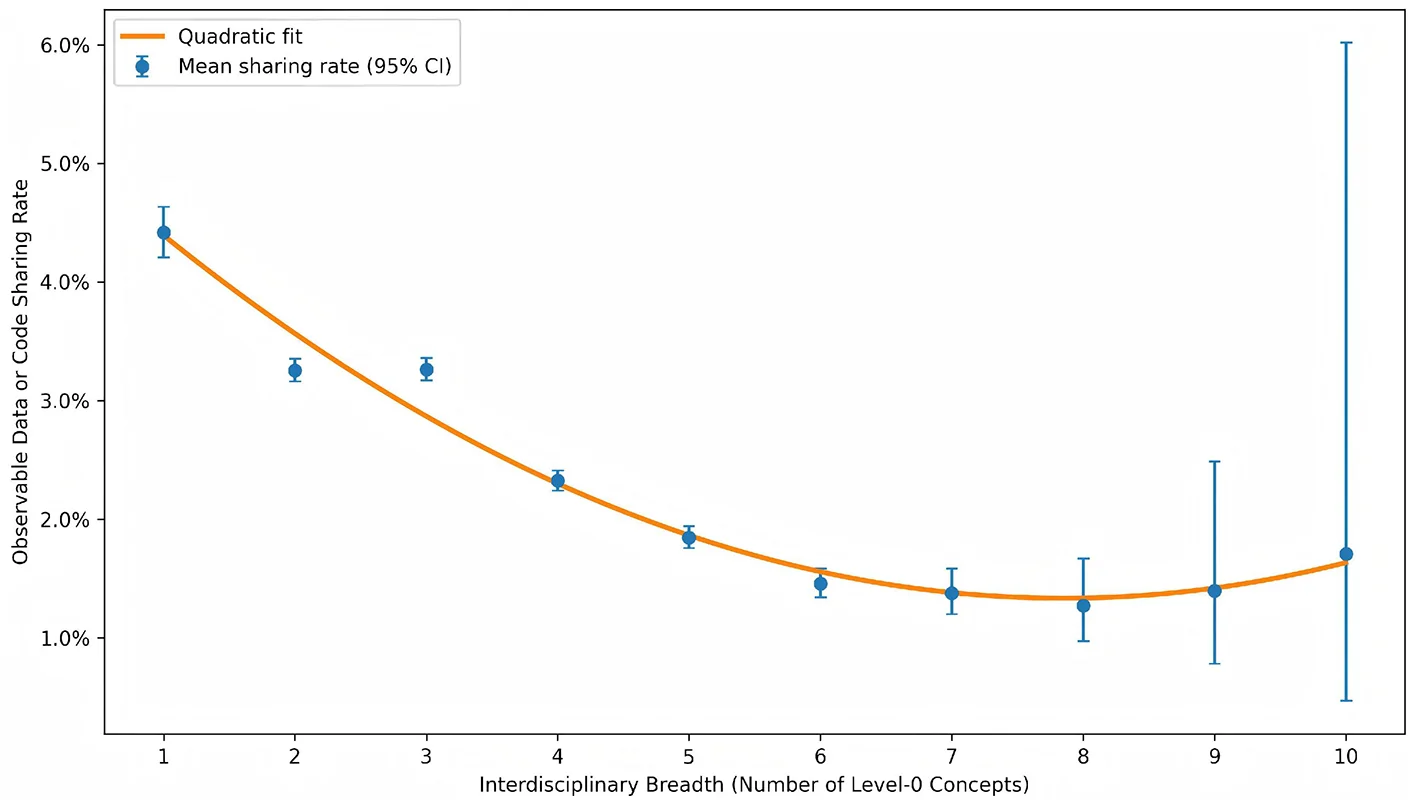
Relationship between interdisciplinary breadth and data-sharing rate.

The LPM further indicated a non-linear association after controlling for publication year and discipline. The linear term was negative (β = −0.0184, robust SE = 0.0007, *p* < 0.001), while the quadratic term was positive (β = 0.00131, robust SE = 0.00008, *p* < 0.001). The estimated turning point was (*Diversity*_*i*_ = 7.04), indicating that the negative association weakened as interdisciplinary breadth increased. However, estimates at the upper end of the breadth distribution were less precise because of the smaller number of observations and wider confidence intervals.

The logistic robustness model produced the same coefficient directions and statistical significance as the LPM. The average marginal effects for interdisciplinary breadth and its squared term were −0.0156 (95% CI: −0.0169 to −0.0143, *p* < 0.001) and 0.00094 (95% CI: 0.00077 to 0.00111, *p* < 0.001), respectively. These estimates were consistent in direction with the LPM results, supporting the robustness of the observed non-linear association.

### 4.4 Disciplinary variation in the citation advantage associated with observable sharing

Table 2 presents the results of the overall OLS model. After accounting for interdisciplinary breadth, publication year, and disciplinary differences, *Share*_*i*_ was positively associated with *Y*_*i*_. The estimated coefficient for *Share*_*i*_ was 0.093 and was statistically significant at the 0.1% level. Converting the coefficient using (*e*^β^−1) × 100 indicates that observable sharing was associated with approximately 9.78% higher citation counts. The corresponding 95% confidence interval ranged from 8.56% to 11.01%. *Diversity* also had a positive and statistically significant coefficient of 0.012, whereas Year_Centered had a negative coefficient of −0.519. The model included disciplinary controls and explained 80.2% of the variation in the log-transformed citation outcome.

**Table 2:** OLS estimates of the overall open data citation advantage.

Variable	Coefficient(β)	Robust standard error
*Share*_*i*_	0.093^***^	0.006
*Diversity*	0.012^***^	0.001
Year_Centered	−0.519^***^	0.001
Discipline controls	Yes	—
Constant	3.493^***^	0.003
*R* ^2^	0.802	—
Observations	566,188	—

^***^p < 0.001.

HC3 heteroskedasticity-robust standard errors are reported.

To assess the influence of the shorter citation window for recent publications, the citation models were re-estimated after excluding articles published in 2025. The overall citation difference associated with observable sharing remained similar at 10.12% [95% CI: 8.98%−11.27%; (*p* < 0.001)], compared with 9.78% in the full 2020–2025 sample. The direction of the discipline-specific estimates also remained unchanged.

Discipline-specific regressions revealed substantial variation in the estimated citation advantage associated with observable sharing. As shown in Table 3, history had the largest positive coefficient, at 0.244, corresponding to an estimated citation difference of 27.67% (95% CI: 23.68%−31.79%). Biology had the second-largest estimate, with a coefficient of 0.176 and a citation difference of 19.25% (95% CI: 15.32%−23.31%). The estimated citation advantages were 11.30% (95% CI: 8.14%−14.55%) in sociology, 9.51% (95% CI: 5.97%−13.17%) in bioinformatics, and 4.19% (95% CI: 2.63%−5.76%) in physics. The magnitude of the positive association therefore varied considerably across these five fields. Digital humanities was the only field with a negative estimate. Its coefficient was −0.222, corresponding to an estimated citation difference of −19.93%. This association was statistically uncertain (*p* = 0.072, 95% CI: −37.14% to 1.99%), with the confidence interval including zero.

**Table 3:** Discipline-specific estimates of the citation advantage associated with observable sharing.

Group	Discipline	Coefficient (β)	Robust SE	Estimated citation difference	95% CI of citation difference
Natural sciences	Biology	0.176^***^	0.0171	19.25%	15.32%−23.31%
Natural sciences	Physics	0.041^***^	0.0077	4.19%	2.63%−5.76%
Humanities and social sciences	History	0.244^***^	0.0162	27.67%	23.68%−31.79%
Humanities and social sciences	Sociology	0.107^***^	0.0147	11.30%	8.14%−14.55%
Interdisciplinary fields	Bioinformatics	0.091^***^	0.0168	9.51%	5.97%−13.17%
Interdisciplinary fields	Digital humanities	−0.222^†^	0.1234	−19.93%	−37.14%−1.99%

^***^p < 0.001.

^†^p < 0.10.

The *p*-value for digital humanities was 0.072.

HC3 heteroskedasticity-robust standard errors and 95% confidence intervals are reported.

### 4.5 Topic-level patterns in history and digital humanities

The discipline-specific regressions showed contrasting patterns in history and digital humanities. History had the largest positive estimate of the open data citation advantage, whereas digital humanities had a negative but statistically uncertain association. BERTopic was therefore applied to all articles in these two fields to examine how data-sharing practices were distributed across their internal topic structures.

#### 4.5.1 History

Figure 4 shows the distribution of data-sharing rates across research topics in history. The topic-level distribution was strongly right-skewed. Most topic clusters were located close to the horizontal axis, indicating sharing rates close to zero. These low-sharing clusters included topics associated with traditional areas of historical research, such as political history, intellectual history, and diplomatic history. In contrast, a small number of topic clusters had substantially higher sharing rates, ranging from approximately 15% to 35%. The higher-sharing topics included clusters labeled with terms related to style transfer and clothing fashion. Their representative terms included references to computational image analysis, art, and fashion history. Other elevated clusters included topics labeled with scientific terminology, such as gauge, Higgs, and molecular. Several quantitatively oriented historical topics also showed relatively high sharing rates. The distribution therefore indicated that the overall sharing in history was unevenly distributed across the field. Higher sharing rates were concentrated in a relatively small number of topics characterized by computational, quantitative, or scientific terminology.

**Figure 4:**
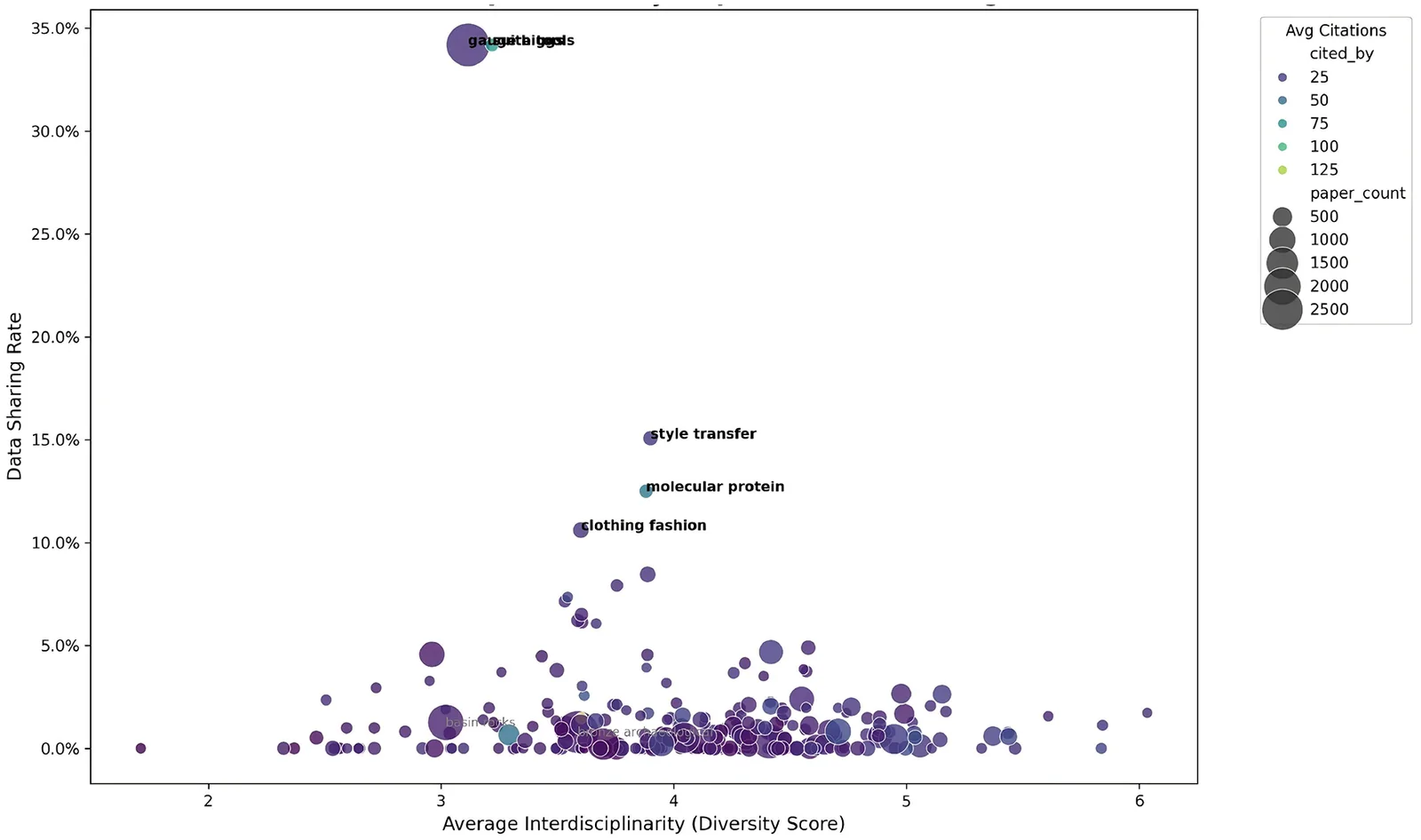
Topic-level distribution of data-sharing rates in history.

#### 4.5.2 Digital humanities

Figure 5 presents the topic-level distribution for digital humanities. Compared with history, the distribution was more dispersed across the semantic space, although most topics still had relatively low sharing rates.

**Figure 5:**
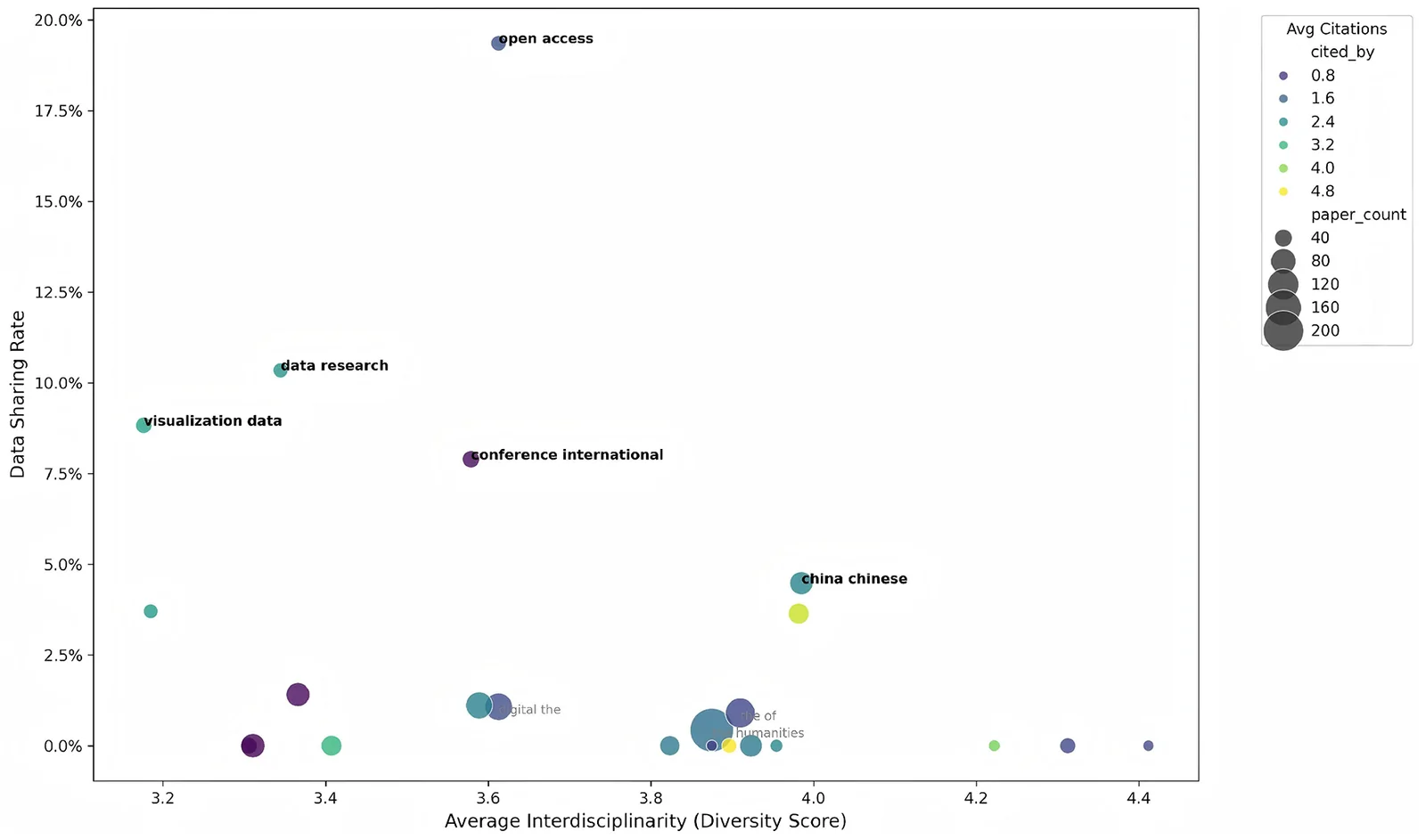
Topic-level distribution of data-sharing rates in digital humanities.

The topic labeled “Open Access” had the highest observed sharing rate. This cluster primarily contained studies concerned with open-access practices and related forms of meta-research. Other topics with relatively high sharing rates included “Visualization Data” and “Data Research,” whose representative terms were related to tools, visualization, data processing, and research infrastructure. A comparatively large topic cluster labeled “Visualization Data” had a sharing rate of approximately 8%−9%, representing a moderate level of data sharing relative to the other digital humanities topics. However, high sharing rates were not broadly distributed across the field. Overall, the topic model indicated that data sharing in digital humanities was concentrated in topics related to open access, methods, tools, and data infrastructure. By contrast, topics oriented toward substantive humanities interpretation generally displayed lower sharing rates.

## 5 Discussion

This study finds several interesting findings. First, interdisciplinary breadth showed a predominantly negative but non-linear association with observable sharing: sharing probability generally declined as breadth increased, although the decline weakened at higher levels. Second, articles with observable sharing evidence were generally associated with higher citation counts, but the magnitude of this citation advantage differed substantially across fields. Third, history and digital humanities showed contrasting patterns: history had the largest positive estimate of the citation advantage, whereas digital humanities showed a negative but statistically uncertain estimate. Topic-level analysis further indicated that observable sharing was unevenly distributed within both fields.

### 5.1 Interdisciplinary breadth and potential challenges for data sharing

The results (Figure 3) showed a predominantly negative but non-linear association between interdisciplinary breadth and observable data sharing. Articles associated with a small number of Level-0 concepts had higher sharing rates, whereas sharing rates generally declined as the number of disciplinary concepts increased, although the decline weakened at higher breadth levels. This finding adds a data-sharing perspective to the literature on interdisciplinarity. Previous studies have mainly examined interdisciplinarity in relation to knowledge integration, collaborative organization, research productivity, and citation impact (Porter et al., 2007; Wagner et al., 2011; Leahey et al., 2017; Hall et al., 2018). The present results suggest that interdisciplinary breadth is also relevant to observable sharing practices.

This pattern is consistent with the concept of data friction, which emphasizes the infrastructural, socio-cultural, and regulatory conditions that shape the movement of data (Bates, 2018; Jeng and He, 2022). Broader disciplinary coverage may involve heterogeneous data formats, metadata standards, ethical conventions, and documentation requirements, increasing the coordination and curation work required to prepare research materials for external use. This interpretation is also consistent with Jeng and He (2022) and Kouper (2023), who highlight the roles of infrastructure, repository arrangements, documentation, and coordination in research data sharing.

The descriptive results also showed that fields conventionally regarded as interdisciplinary did not necessarily have the highest values of interdisciplinary breadth. Bioinformatics, for example, had the lowest mean interdisciplinary breadth (2.34) among the six fields, despite being widely understood as an interdisciplinary field. This finding suggests that interdisciplinary breadth, as operationalized through the number of Level-0 OpenAlex concepts, captures the range of disciplinary domains associated with an article. A field may be interdisciplinary in origin while remaining concentrated around a relatively stable combination of core disciplines. This distinction is consistent with the broader literature showing that interdisciplinarity is multidimensional and cannot be represented fully by a single indicator (Porter et al., 2007; Wagner et al., 2011). In the present study, interdisciplinary breadth captures the variety of top-level disciplinary concepts associated with an article, but does not directly represent their balance, cognitive distance, or depth of integration. The comparatively low mean score for bioinformatics should therefore be interpreted within the limits of this operationalization.

### 5.2 Disciplinary variation in the open data citation advantage

The overall regression results indicated that observable sharing was associated with approximately 9.78% higher citation counts after accounting for interdisciplinary breadth, publication year, and disciplinary differences. This result is consistent with previous studies reporting a positive relationship between data sharing and citation impact (Piwowar and Vision, 2013; Colavizza et al., 2020, 2024). Previous studies have reported varying magnitudes of citation advantage across datasets and definitions of sharing, placing the present estimate within a broader pattern of positive associations. The estimate remained similar after articles published in 2025 were excluded, suggesting that the overall association was not driven by the shorter citation window of the most recent publication cohort (Piwowar and Vision, 2013; Colavizza et al., 2020, 2024). The present study extends this literature by showing that the magnitude of this association varies substantially across research fields.

One possible explanation is that accessible research materials can improve transparency, discoverability, and opportunities for reuse. Articles linked to accessible datasets or code may therefore provide additional value beyond the published text itself. At the same time, the present observational design does not establish that data sharing alone produced the citation difference. Articles produced by better-resourced teams, published in more visible venues, or based on more reusable research materials may also be more likely both to share data and to receive citations.

At the field level, history showed the largest positive estimate, followed by biology, sociology, bioinformatics, and physics. This pattern suggests that the scholarly returns associated with data sharing may vary across disciplinary contexts, including research practices, data characteristics, and existing openness norms.

The comparatively small estimate in physics may be related to the normalization of open practices in parts of the field. Where data sharing, code release, or large-scale collaborative infrastructures are already relatively common, sharing may provide less additional visibility because it is less distinctive. In such contexts, data availability may function as an expected component of research practice. This interpretation is consistent with recent discussions of data-sharing practices in physics, which highlight substantial disciplinary variation in how openness is integrated into research workflows (Nature Publishing Group, 2023). The larger estimate in history may be related to the relative scarcity of structured and reusable datasets, although the present analysis does not directly test this mechanism (Higgins et al., 2024; Farina et al., 2025).

### 5.3 Contrasting patterns in history and digital humanities

History and digital humanities presented the most contrasting discipline-specific estimates. History showed a citation advantage of 27.67% for articles with observable sharing evidence, whereas digital humanities showed an estimated citation difference of −19.93%. However, the digital humanities estimate had a *p*-value of 0.072 and a 95% confidence interval that included zero, indicating substantial statistical uncertainty. The comparison below is therefore exploratory and provides possible interpretations of the observed topic-level patterns.

The topic-level analysis showed marked within-field variation in both disciplines. In history, data sharing was highly concentrated in a small number of computational, quantitative, or dataset-intensive topics. Most historical topics had sharing rates close to zero, whereas a limited number of clusters involving image analysis, quantitative approaches, or scientific terminology displayed substantially higher rates. One possible interpretation is that structured and reusable historical datasets remain relatively uncommon, so studies that provide such resources may be more visible within particular research areas. This interpretation is consistent with recent discussions of humanities data practices, which emphasize that historical and cultural data often require substantial organization, documentation, and curation before they can become reusable research resources (Higgins et al., 2024; Farina et al., 2025).

The digital humanities pattern was different. Higher sharing rates were concentrated in topics related to open access, visualization, tools, data processing, and research infrastructure. This concentration suggests that observable sharing was particularly common in method-, tool-, and infrastructure-oriented topics. A possible interpretation of the negative estimate is that shared outputs in digital humanities—including datasets, software, databases, digital editions, and visualization tools—may receive recognition through channels that are only partly reflected in article-level citations. Such outputs may be reused without being consistently acknowledged through conventional journal-article references. This issue has also been discussed in broader debates on recognizing digital scholarly outputs, where datasets, software, and other research objects are increasingly considered scholarly contributions but remain unevenly incorporated into traditional evaluation systems (All European Academies, 2023).

Interoperability may also matter. Digital humanities projects often adapt metadata schemas, encoding standards, database structures, and software environments to specific collections or research questions (Belteki et al., 2025; Farina et al., 2025). Such customization can complicate reuse across projects. Data- and tool-oriented outputs may also receive uneven recognition in evaluation systems centered on conventional publications (Farina et al., 2024). These explanations remain tentative because the topic model identifies patterns of variation and does not directly test scarcity, interoperability, or recognition mechanisms. The comparatively small digital humanities sample and the statistically uncertain citation estimate also warrant cautious interpretation.

### 5.4 Implications for research data-sharing support

The findings have several implications for the design of research data-sharing support. These implications can be organized around key stakeholders in the data-sharing process, including data-sharing platforms and repositories, disciplinary associations and research institutions, and publishers and evaluation systems. For data-sharing platforms and repositories, support could account for the additional coordination and documentation requirements of research spanning multiple disciplinary domains. Uniform requirements may be difficult to implement when a single project combines materials governed by different technical, ethical, and disciplinary standards. Platforms and repositories could therefore provide greater support for compatibility across disciplinary metadata practices, including metadata mapping and documentation, which may reduce some of the work involved in preparing heterogeneous materials for deposit and reuse.

For disciplinary associations, funding agencies, and research institutions, discipline-sensitive support may also be useful. The relatively low sharing rates in history and sociology suggest that infrastructure alone may not be sufficient. Researchers may also need guidance on documenting contextualized, copyrighted, ethically sensitive, or interpretive materials. Data-sharing support could therefore distinguish between research materials that can be made openly downloadable and those that require restricted, mediated, or conditional access. Guidance on repository selection may also help researchers identify suitable arrangements for different types of research materials.

For publishers and evaluation systems, the disciplinary variation in citation associations suggests that systems of scholarly recognition may value data-related outputs differently across fields. Journals and evaluation systems could support data sharing by providing clearer recognition for datasets, software, databases, and digital collections through data-paper formats, dedicated publication venues, and more explicit data-citation practices. Distinguishing article-level impact from the use and citation of associated research objects may help capture forms of scholarly contribution that are not fully reflected in conventional article citations.

### 5.5 Limitations and future research

This study has several limitations. First, the disciplinary classification and interdisciplinary breadth measurement were based on OpenAlex concepts. Although this approach enables large-scale analysis, algorithmically generated concepts may not fully capture the substantive boundaries or knowledge relationships of individual studies. Future research could incorporate expert-based classification or reference-based diversity measures that consider disciplinary distance and knowledge integration. In addition, the sampling procedure ranked articles by citation count within each discipline-year stratum before retrieving up to 20,000 records. For strata that exceeded this limit, the retained records were therefore skewed toward more highly cited articles. This sampling strategy was adopted to maintain comparability across disciplines and publication years and to enable large-scale article-level and text-based analyses. However, it may reduce the representativeness of the sample with respect to the full OpenAlex publication population. Therefore, the findings should be interpreted as evidence from a structured comparative sample rather than direct estimates of patterns across all publications. Future studies could incorporate complete disciplinary samples or probability-based sampling strategies to further examine the generalizability of these findings.

Second, the identification of observable sharing practices relied on observable signals, including repository links, supplementary materials, and textual sharing statements. Because the binary indicator combined evidence of data and code availability, it should be interpreted as a measure of observable research-material sharing, although data-sharing records constituted the large majority of identified cases. This approach may not fully capture the accessibility, documentation quality, interoperability, or reuse potential of shared resources. The rule-based identification procedure was also not independently validated against a manually coded subsample, so some misclassification may remain. Future studies could combine automated identification with manual validation and distinguish more precisely among open data, controlled-access data, code sharing, and other forms of research-material availability.

Third, this study was based on observational bibliometric data, and the regression models identify associations. Unobserved characteristics of articles, authors, journals, or research teams may influence both sharing decisions and citation outcomes. Future research using longitudinal designs or quasi-experimental approaches could further examine causal mechanisms. Although publication year was controlled in the models and the main citation results remained similar after excluding 2025 articles, differences in citation accumulation time cannot be fully eliminated because newer publications inherently have shorter exposure periods for accumulating citations in bibliometric analyses.

Fourth, citation counts provide an incomplete representation of research impact, particularly in fields where books, databases, software, and digital resources are important scholarly outputs. In addition, the relatively small digital humanities sample and the statistical uncertainty of its citation pattern require further validation with larger and more comprehensive datasets. Future research could also examine direct citations to datasets or software, downloads, reuse events, and other indicators of the impact of shared research objects.

## 6 Conclusion

This study investigated how interdisciplinary breadth, observable sharing practices, and citation impact were associated across different disciplinary contexts. The results showed that interdisciplinary breadth had a predominantly negative but non-linear association with observable sharing, with sharing probability generally declining as breadth increased, although the decline weakened at higher levels. Articles with observable sharing evidence were generally associated with higher citation counts, but the magnitude and direction of this association varied across disciplines. History showed the largest positive estimate of the open data citation advantage, whereas digital humanities showed a negative but statistically uncertain association. Topic-level analysis suggested that observable sharing in history was concentrated in a small number of computational and dataset-intensive topics, while sharing in digital humanities was more closely associated with open-access, tool-oriented, and infrastructure-related research. Policies intended to promote research data sharing could therefore take into account differences in data types, documentation requirements, ethical constraints, and forms of scholarly contribution. More flexible data-sharing support, including appropriate documentation and infrastructure, may help accommodate heterogeneous research materials across disciplinary settings.

## Data Availability

The original contributions presented in the study are included in the article/supplementary material, further inquiries can be directed to the corresponding author.
